# ACSA2 is not astrocyte-specific: implications for cell sorting strategies in the rodent brain

**DOI:** 10.3389/fncel.2026.1756677

**Published:** 2026-02-18

**Authors:** Emerson Daniele, Gabriel Khelifi, Daniel Beretta, Boyan K. Tsankov, K. W. Annie Bang, Chiara Beretta, Dana J. Philpott, Samer M. Hussein, Maryam Faiz

**Affiliations:** 1 Institute of Medical Science, University of Toronto, Toronto, ON, Canada; 2 Department of Molecular Biology, Medical Biochemistry and Pathology, Faculty of Medicine, Université Laval, Québec City, QC, Canada; 3 Oncology Division, CHU de Québec-Université Laval Research Centre, Québec City, QC, Canada; 4 Université Laval Cancer Research Centre, Québec City, QC, Canada; 5 Department of Cell Biology and Anatomy, Hotchkiss Brain Institute, University of Calgary, Calgary, AB, Canada; 6 Department of Immunology, University of Toronto, Toronto, ON, Canada; 7 Network Biology Collaborative Centre Flow Cytometry Facility, Sinai Health, Lunenfeld-Tanenbaum Research Institute, Toronto, ON, Canada; 8 Alberta Children’s Hospital Research Institute, University of Calgary, Calgary, AB, Canada; 9 Hotchkiss Brain Institute, University of Calgary, Calgary, AB, Canada; 10 Department of Laboratory Medicine and Pathobiology, University of Toronto, Toronto, ON, Canada; 11 Division of Anatomy, Department of Surgery, University of Toronto, Toronto, ON, Canada

**Keywords:** ACSA2, astrocyte, brain cell isolation, FACS, MACS, scRNA-seq

## Abstract

**Introduction:**

Astrocyte-specific cell surface antigen-2 (ACSA2) has been established as the gold-standard marker for isolating astrocytes via magnetic-activated cell sorting (MACS) or fluorescence-activated cell sorting (FACS) for downstream transcriptomic studies. In a prior study of the astrocyte response to cortical stroke, we used ACSA2-based cell sorting prior to single cell RNA sequencing (scRNAseq). We found a substantial population of ACSA2^+^ cells exhibiting robust microglial gene expression signatures, suggesting contamination of purified astrocyte preparations.

**Methods:**

An intravenous antibody labeling strategy coupled with flow cytometry was used to determine whether contamination originated from circulating immune cells or microglia.

**Results:**

Contaminating cells were identified as CNS-resident microglia that express CD45, CD11b, and ACSA2.

**Discussion:**

These findings caution against the usage of ACSA2 for astrocyte purification without exclusion markers to achieve high-purity astrocyte populations for downstream multi-omics analyses.

## Introduction

Astrocytes play critical roles in maintaining homeostasis and contribute to neuroinflammatory responses to CNS injury and disease (Escartin et al., 2021; Hasel and Liddelow, 2021; Sofroniew and Vinters, 2010). Understanding astrocyte biology at the molecular level requires the isolation of pure astrocyte populations for downstream transcriptomic, proteomic, and epigenomic analyses. However, recent reports have raised concerns about immune cell contamination of “pure” astrocyte populations isolated from CNS tissue (Lee et al., 2024; O’Dea and Liddelow, 2025).

Astrocyte-cell-specific-antigen-2 (ACSA2), also known as ATP1B2, is expressed on the surface of astrocytes. Consequently, ACSA2-based fluorescence-activated cell sorting (FACS) and/or magnetic-activated cell sorting (MACS) strategies (Batiuk et al., 2017; Kantzer et al., 2017) have been widely adopted for the study of astrocytes in both health (Batiuk et al., 2020) and disease (Figueroa et al., 2025; Moreno-García et al., 2020). Despite widespread use, the potential for immune cell contamination in ACSA2-sorted populations has not been investigated. Microglia are CNS-resident immune cells expressing CD45, CD11b, CX3CR1, and P2RY12 (Gerrits et al., 2020; Holtman et al., 2015). Previous studies using ACSA2 relied on CD11b to assess microglial contamination (Batiuk et al., 2017), however, surface expression of CD11b can be variable following tissue dissociation (Mattei et al., 2020). Therefore, CD11b may not reliably capture all immune cell populations identified by the pan-immune cell marker, CD45 (Donovan and Koretzky, 1993). Consistent with these limitations, analysis of a single cell RNA sequencing (scRNAseq) dataset comprized of ACSA2^+^ sorted cells from the uninjured and stroke-injured mouse brain showed microglial gene expression (Scott et al., 2024). However, microglial expression of ACSA2 has not been directly assessed.

Here, we verify that microglia can express *Atp1b2*, the gene encoding ACSA2. Using an *in vivo* CD45 antibody labeling strategy via intravenous injection, we confirm that these contaminating cells originate from the CNS parenchyma rather than the circulation. We further validate their identity as microglia through flow cytometric phenotyping with antibodies against Cd11b and CD45. These findings highlight the need for refined gating strategies in ACSA2-based sorting protocols and stringent cell type annotation methods to exclude immune cells from downstream molecular analyses.

## Materials and methods

### Animals

P56 male C57BL/6N mice were obtained from Charles River Laboratories and housed in Allentown cages with corncob bedding, paper nestlets, and environmental enrichment with *ad libitum* access to rodent chow (Envigo, cat# 2918) and non-acidified water. Mice were maintained in a temperature- and humidity-controlled room on a 12:12 light-dark cycle. All animal procedures were conducted under approved animal use protocol 20013084 at the Division of Comparative Medicine (DCM), University of Toronto.

### CD45 intravenous labeling

Mice received an intravenous tail vein injection of 3 μg anti-CD45.2-PE antibody (clone 104, BioLegend, cat# 109808) or an isotype control, PE rat IgG2b, κ antibody (Biolegend, cat# 400608) diluted in sterile PBS (Anderson et al., 2014). After 3 min to allow antibody circulation and labeling of intravascular leukocytes, mice were euthanized via CO2 asphyxiation and brain tissue was rapidly harvested and transferred into a petri dish containing cold calcium/magnesium-free DPBS (Wisent, MultiCell, cat# 311-425) prior to issue dissociation.

### Tissue dissociation

The olfactory bulb and cerebellum were removed, and brain tissue was washed in calcium/magnesium-free DPBS (Wisent, MultiCell, cat# 311-425), prior to dissociation with the Adult Brain Dissociation Kit (P) kit (Miltenyi Biotec, cat# 130-107-677). GentleMACS Octo Dissociator (Miltenyi Biotec, cat# 130-134-029) settings were based on those used by Liu et al. (2021). Following dissociation, sample cleanup was performed in accordance with the manufacturers protocols for: (i) Debris Removal Solution (Miltenyi Biotec, cat# 130-109-398), (ii) Myelin Removal Beads II (Miltenyi Biotec, cat#130-096-733), or (iii) Debris Removal followed by Myelin Removal. The resulting cell pellet was then processed for flow cytometry.

### Flow cytometry

Cells were incubated in blocking buffer comprised of rat anti-mouse CD16/CD32 (1:100, Mouse BD Fc Block, BD Biosciences, clone 2.4G2, cat# 553141) and 0.5% BSA (BioShop, cat: ALB005.100) in calcium/magnesium free DPBS (Wisent, MultiCell, Cat: 311-425) for 20 min at 4 °C. For IV labeling experiments, cells were subsequently stained with the following antibodies diluted in blocking buffer for 15 min at 4 °C; anti-ACSA2-APC (1:200, Miltenyi Biotec, clone REA969, cat# 130-117-535) and anti-CD45-BV605 (1:200, BioLegend, clone 30-F11, cat# 103140). For microglial phenotyping, cells were subsequently stained for 15 min at 4 °C with the following antibodies diluted in blocking buffer: anti-CD45-BV605 (1:200, BioLegend, clone 30-F11, cat# 103140), anti-CD11b-VioBrightFITC (1:200, Miltenyi Biotec, Clone REA592, 130-113-805) and anti-ACSA2-APC (1:200, Miltenyi Biotec, clone REA969, cat# 130-117-535). Cells were washed twice in FACS buffer (DPBS + 0.5 % BSA) and centrifuged for 7 min at 4 °C and 300 × *g*. Prior to acquisition, cell viability was assessed using DAPI (Thermo Scientific, cat# 62248), and nucleated cells were identified with DRAQ5 (Thermo Scientific, cat# 62254). Flow cytometry was performed on a BD FACSDiscover S8 (Network Biology Collaborative Centre, Toronto, ON, Canada) and data were analyzed using FlowJo (v 10.8.1, BD Biosciences).

### Immunohistochemistry

Mice were euthanized via transcardiac perfusion with 30 mL ice-cold DPBS followed by 30 mL of 4% (w/v) paraformaldehyde (PFA, BioShop, cat# PAR070) under isoflurane anesthesia. Brains were harvested and post-fixed in 4% PFA overnight, then transferred to 30% (w/v) sucrose solution for cryoprotection. Brains were sectioned in series at 20 μm thickness at −25 °C using a cryostat (Thermo Scientific, HM525 NX). Sections were collected onto SuperFrost Plus microscope slides (Fisherbrand, cat# 1255015) and stored at −20 °C until staining.

Sections were rehydrated with three washes of DPBS, then incubated in blocking buffer containing 5% BSA (BioShop, cat#ALB001) and 0.1% Tween-20 (Bioshop, cat# TWN510) for 1 h at room temperature in a humidified chamber. Slides were incubated overnight at 4 °C in primary antibody solution containing anti-TMEM119 (1:100; Abcam, cat# AB209064) in a humidified chamber. Following three DPBS washes, slides were incubated for 1 h at room temperature with donkey anti-rabbit Alexa Fluor 647 (1:400, Invitrogen, cat#A31573) and anti-ACSA2-PE (1:50; Miltenyi Biotec, cat# 130-116-244, clone REA969). Tissue was washed three times in DPBS, counterstained with DAPI (1:3000; Invitrogen, cat# D3571), and mounted with Mowiol (Sigma-Aldrich, cat# 81381) mounting medium.

Images were acquired using a 20 × objective on a Zeiss LSM 900 confocal microscope with Zen Blue software (version 3.3; Zeiss). Intensity thresholds were set based on background signal measured in secondary-only and a rat IgG2b-PE isotype control (1:50, Miltenyi Biotec, Cat# 130-123-273). Linear brightness adjustments and colocalization analysis of TMEM119 and ACSA2 were performed using Zen Blue.

### Single cell bioinformatics

Raw single cell RNA seq reads in fastq format were downloaded from SRA (BioProject: PRJNA952594, Runs: SRR27010906 and SRR27010907). Reads were aligned to the mouse mm10/GRCm38 genome, demultiplexed and counted with the 10x Genomics Cell Ranger v8.0.1 using default parameters and the Ensembl mouse transcriptome release 99. Demultiplexed counts matrices where then processed in R version 4.5.1 using Seurat v5.3.0 (Butler et al., 2018; Hao et al., 2021, 2024; Rosenberg et al., 2018; Satija et al., 2015; Stuart et al., 2019). Cells were merged as a single Seurat object. A filter specified a minimum of 200 features per cell and 10 cells per feature from each set of samples. Data was processed using Seurat’s recommended sctransform workflow: first sctransform was applied to normalize feature counts; followed by PCA and UMAP dimension reductions; followed by finding neighbors; then finding clusters and their respective markers. The log-normalized counts were scaled gene-wise from a range of 0 to 1 across cell-types, to generate heatmaps with the ComplexHeatmaps R package v2.24.1 (Gu et al., 2016) and visualize expression of astrocyte and microglia features. The following R packages were also used for visualization or processing: ggplot2 (Wickham, 2016), dplyr (Wickham et al., 2025), circlize (Gu et al., 2014) and Viridis (Garnier et al., 2024).

The MapMyCells: Cell Type Mapper v1.6.1 library from Allen Institute [RRID:SCR_024672] was used with the Hierarchical method in Python 3.13.8, with precomputed statistics to annotate cell types for each cell. 10x Whole Mouse Brain taxonomy (CCN20230722) rev 230821 was used as reference. Seurat object data was exported for the cell type mapping to maintain consistency between processing steps. The cell annotations were in turn used to count proportion of cell types (Supplementary Table 1) in a bar plot and apply categorization in a dim plot.

Module scores showing relative gene expression of astrocyte and microglial marker genes (Supplementary Table 2) were calculated using Seurat’s AddModuleScore, using default settings.

The source code is available on a Git repository^1^, where the release tag published marks the revision that generated the figures in this paper. Exact versions of all used software packages can be automatically retrieved using the lock files in the code repository.

## Results

### scRNA-seq reveals contamination in ACSA2-sorted cells from naïve and stroke-injured mouse cortices.

**FIGURE 1:**
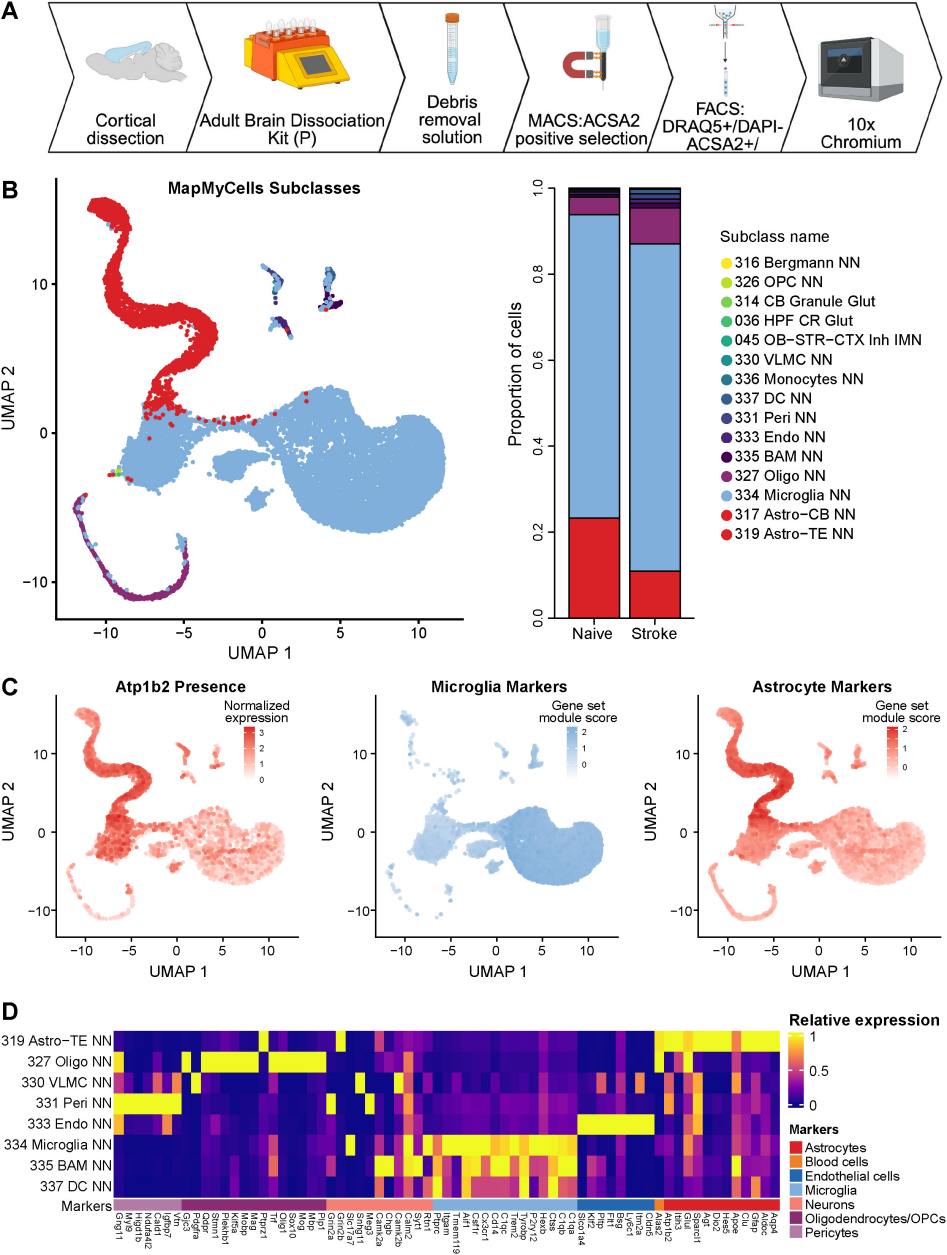
Single-cell analysis of ACSA2-enriched populations in stroke and uninjured conditions. **(A)** Schematic demonstrating the purification of ACSA2 positive cells by MACS and FACS for downstream single cell RNA sequencing using the 10x Chromium platform in Scott et al. (2024). Created in BioRender. Daniele, E. (2026) https://BioRender.com/gszrag9. **(B)** UMAP visualization of merged stroke (6,889 total cells) and naïve (9,856 total cells) scRNA-seq datasets from ACSA2-sorted cells, with MapMyCells cell type annotations (left) and cell type proportions comparing stroke versus naïve conditions in ACSA2-sorted fractions (right). Astro-TE, astrocytes-telencephalon, Oligo, oligodendrocytes, VLMC, vascular leptomeningeal cells, Peri, pericytes, Endo, endothelial cells, BAM, border-associated macrophages, DC, dendritic cells, NN, non-neuronal. Full annotation abbreviations can be found here: MapMyCells Abbreviations. **(C**) Feature plots showing normalized expression of *Atp1b2* (gene encoding ACSA2) and modules scores for microglia and astrocyte marker genes in the merged dataset. **(D)** Heatmap displaying relative expression of canonical cell type markers across annotated cell clusters, confirming cluster identities and expression of *Atp1b2* expression in microglia. Data from Scott et al. (2024).

To assess the purity of ACSA2-sorted astrocyte preparations used in downstream transcriptomics analyses, we reanalyzed a previously published 10x Genomics Chromium scRNAseq dataset of ACSA2-MACS-FACS-purified astrocytes from the murine stroke-injured and naïve cortex (Figure 1A; Scott et al., 2024). Using the Allen Institute’s MapMyCells [RRID:SCR_024672], we classified astrocytes, microglia, border-associated macrophages (BAMs), oligodendrocyte progenitor cells (OPCs), endothelial cells, pericytes, dendritic cells (DCs), and vascular leptomeningeal cells (VLMCs). We next investigated the proportion of cell types across stroke and naïve datasets and observed that microglia represented 53% and 76% of cells in the dataset, respectively (Figure 1B and Supplementary Table 1). When we examined the relative gene expression of canonical cell type genes, *Atp1b2*, the gene encoding ACSA2, was highly enriched in astrocytes (Figures 1C, D), however, lower gene expression was still observed in microglia, suggesting that these cells are present within the ACSA2 sorted fractions.

### Immune contamination of the ACSA2^+^ fraction are CNS-resident microglia

**FIGURE 2:**
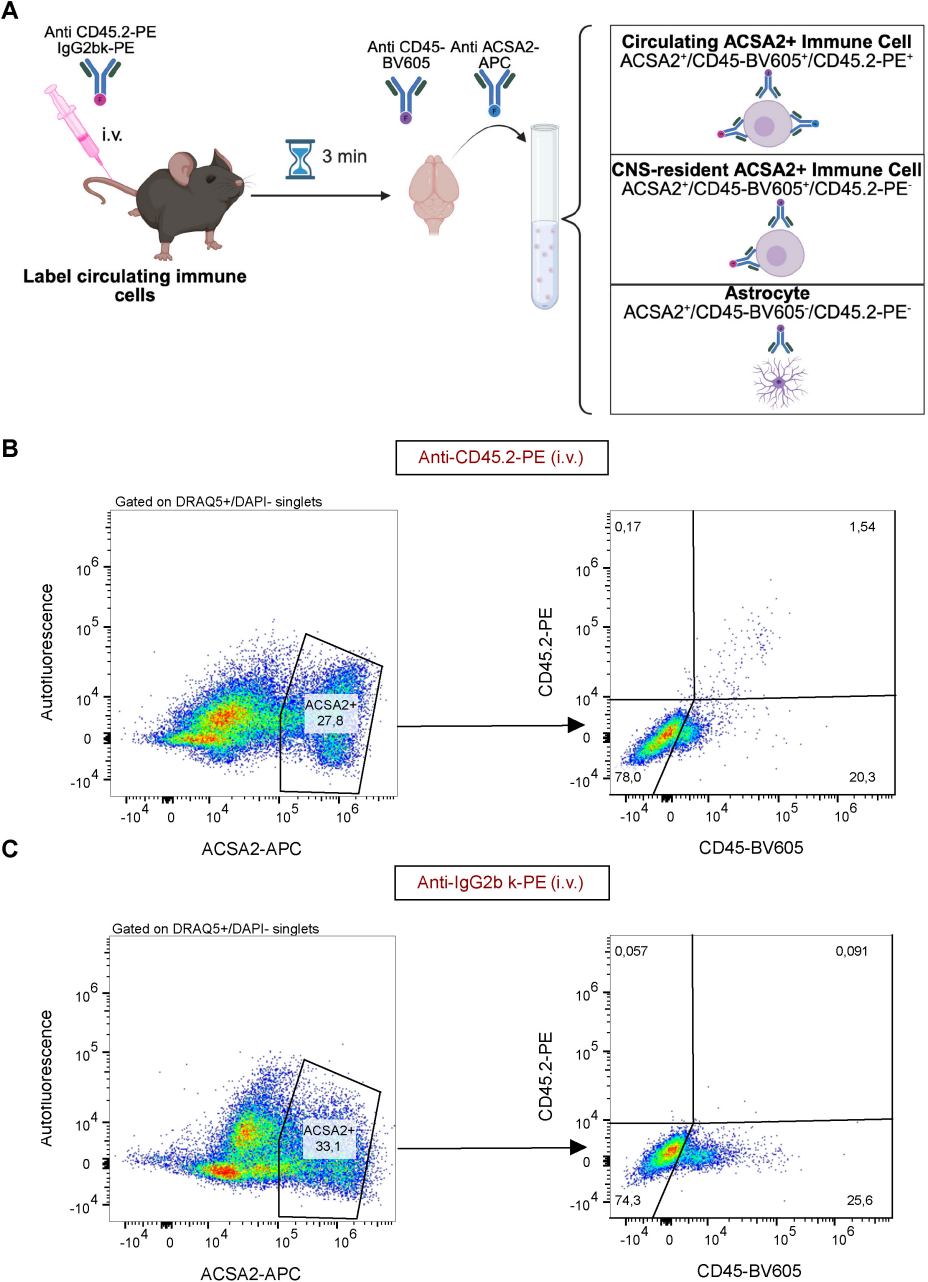
Intravenous antibody labeling distinguishes CNS-resident from circulating immune cells in ACSA2^+^ fractions. **(A)** Mice received I.V. injection of fluorescent antibody 3 min before tissue harvest and brain dissociation. I.V.-labeled cells represent circulating immune cells, while unlabeled CD45^+^ cells are CNS-resident. Created in BioRender. Daniele, E. (2026) https://BioRender.com/20anz0d. **(B)** Flow cytometry using anti-CD45.2-PE (I.V.) shows 20.3% of ACSA2^+^ cells are CD45-BV605^+^/CD45.2-PE^–^, indicating CNS-resident immune cell contamination, while only 1.54% are circulating immune cells (CD45-BV605^+^/CD45.2-PE^+^). **(C)** Isotype control using IgG2b-κ-PE (I.V.) shows minimal non-specific labeling confirming specificity of CD45.2 labeling. Gates set on live, nucleated DRAQ5+/DAPI- singlets. Representative plots from *n* = 3 mice per condition.

To assess whether ACSA2 was expressed on the surface of microglia, we performed flow cytometric analysis. To distinguish microglia from circulating myeloid cells that can also express ACSA2, we performed an intravenous tail vein injection with anti-CD45.2-PE antibody 3 min prior to tissue harvest, followed by *ex vivo* staining with antibodies against CD45-BV605 and ACSA2-APC (Figure 2A). This approach distinguishes intravascular leukocytes (labeled with I.V. antibody; CD45.2-PE+) from tissue-resident cells (unlabeled; CD45.2-PE-), while the *ex vivo* CD45-BV605 staining labels all CD45^+^ cells regardless of location. Using this strategy, we identified two populations of ACSA2^+^ cells: circulating ACSA2^+^ immune cells (CD45.2-PE^+^/CD45-BV605^+^/ACSA2-APC^+^) and CNS-resident ACSA2^+^ immune cells (CD45.2-PE^–^/CD45-BV605^+^/ACSA2-APC^+^) (Figure 2A). Quantification revealed that CNS-resident ACSA2^+^ immune cells comprised 26.3 ± 3% (*n* = 3) of all live, nucleated, ACSA2^+^ cells in the brain. Circulating ACSA2^+^ immune cells constituted 2.03 ± 0.3% (*n* = 3) of the total ACSA2^+^ population (Figures 2B, C), which is consistent with the observation of DCs in our scRNAseq data (Figure 1B). These findings demonstrate that CNS-resident ACSA2^+^ cells are the predominant source of contamination.

**FIGURE 3:**
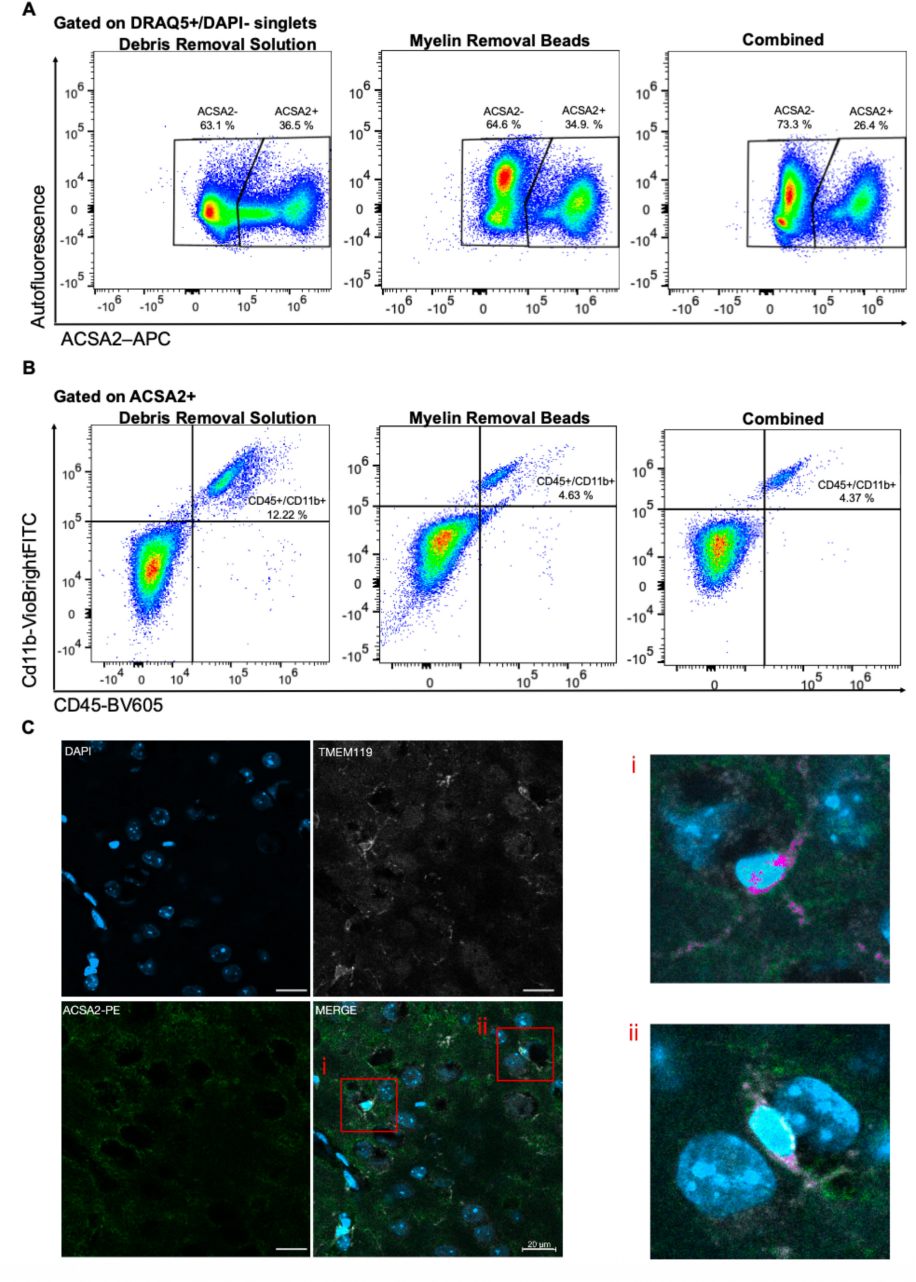
CD45^+^/CD11b^+^ microglia are present in ACSA2^+^ astrocyte-enriched fractions across debris removal methods. **(A)** Representative dot plots show ACSA2+ and ACSA2– live, nucleated DRAQ5+/DAPI- singlets following debris removal solution, myelin removal beads, and combined approaches. **(B)** Representative dot plots show CD45 and CD11b expression in ACSA2+ gated populations. **(C)** Representative images of DAPI+ (nuclear, blue), TMEM119+ (microglia marker, white), and ACSA2-PE+ (astrocytes, green) cells in the naïve adult mouse cortex. Higher magnification of boxed cells (I, ii) show co- localization (fuchsia) of TMEM119+ microglia with ACSA2+ signal. Scale bar = 20 μm.

To validate the microglial identity of ACSA2^+^ CNS-resident cells, we examined the expression of CD45, CD11b, and ACSA2 by flow cytometry. Debris removal is a critical step in CNS tissue processing, and previous studies employing ACSA2-based isolation have utilized either debris removal solution or myelin removal beads (Batiuk et al., 2017; Scott et al., 2024). We therefore assessed whether these strategies contribute to different rates of microglial contamination of ACSA2^+^ fractions. Debris removal solution resulted in 15.2 ± 3.2% (*n* = 3) of ACSA2^+^ cells that were CD45^+^/CD11b^+^ microglia (Figures 3A,B). Myelin removal beads reduced this contamination to 3.9 ± 0.5% (*n* = 3). Combining these two strategies provided no additional reduction in microglia (5.5 ± 0.9%, *n* = 3) (Figures 3A,B). The majority of CD45^+^/CD11b^+^ cells were found in the ACSA2- fraction across all three methods (∼78%–82%; Supplementary Figure 1). To note, while debris removal solution and myelin removal beads alone yielded similar proportions of ACSA2^+^ cells (34.8 ± 2.1% and 35.8 ± 0.6%, respectively), the combined approach reduced ACSA2^+^ yield to 24.4 ± 1.6% without a corresponding reduction in microglia contamination. To confirm the microglial identity of these contaminating cells *in situ*, we performed immunohistochemistry on adult brain tissue and observed ACSA2^+^/TMEM119^+^ cells (Figure 3C). Altogether, these data suggest that a subset of microglia express ACSA2.

## Discussion

In this report, we examined microglial expression of ACSA2. First, we used a published scRNAseq dataset of ACSA2-sorted cells isolated by MACS and FACS purification from the murine naïve and stroke-injured cortex (Scott et al., 2024). In this dataset, we found that microglia express *Atp1b2.* Flow cytometry analysis confirmed that CD45^+^CD11b^+^ microglia are a major contaminant in ACSA2^+^ populations. We validated this finding *in situ*, observing co-localization of ACSA2 with TMEM119, a marker of homeostatic microglia, in the naïve adult mouse brain.

Reanalysis of scRNAseq data showed that in addition to microglial contamination, oligodendrocyte lineage cells and endothelial cells are present in the ACSA2 sorted fraction, as previously characterized (Batiuk et al., 2017). We also identified dendritic cells (DCs) and border associated macrophages (BAMs) as minor but previously unrecognized contributors to non-astrocyte populations in ACSA2-sorted samples. Using MapMyCells, we observed *Atp1b2* expression in microglia, albeit at a relatively lower expression level than astrocytes. Taken together, these observations highlight the importance of stringent cell type annotation in single-cell RNA sequencing workflows.

Batiuk et al. (2017), Kantzer et al. (2017) characterized ACSA2 as an astrocyte-specific surface marker for purification by MACS or FACS. While our study used similar dissociation protocols, differences in debris removal steps led to differences in contamination (Table 1). Batiuk et al. (2017), demonstrated that combined Percoll Plus and myelin bead removal achieves high purity (0.2 % CD11b+ contamination), in ACSA2 sorted populations while myelin bead removal alone results in 29 % ACSA2+/Aldh1l1-eGFP- cells in unsorted preparations, indicating that not all ACSA2+ cells express the astrocyte lineage reporter. Scott et al. (2024) further demonstrated the importance of debris removal methods whereby debris removal solution yielded substantial microglial contamination by scRNAseq, whereas myelin bead removal achieved negligible contamination (Table 1).

**TABLE 1:** Summary of debris removal and resulting contamination in ACSA2 populations.

Study	Analysis method	Tissue condition	Debris removal method	Contamination
Batiuk et al., 2017	ACSA2 MACS, flow cytometry (ACSA2,CD11b)	P56, naïve	Percoll plus and Myelin bead removal	0.2 % (ACSA2+/CD11b^+^)
Batiuk et al., 2017	Unsorted, flow cytomety (ACSA2, Aldh1l1-eGFP reporter)	P56, naïve	Myelin bead removal	29 % of ACSA2+ (eGFP-)
Scott et al., 2024	ACSA2-MACS-FACS, 10X Chromium	D10 post-stroke	Myelin bead removal	Negligible
Scott et al., 2024	ACSA2-MACS-FACS, 10X Chromium	Naïve and D10 post-stroke	Debris removal solution	53 % (Naïve) 76 % (Stroke)
Current study	Unsorted, flow cytometry (CD45, Cd11b, ACSA2)	P56, naïve	Debris removal solution	15 % (ACSA2^+^/CD45^+^/CD11b^+^)
Current study	Unsorted, flow cytometry (CD45, Cd11b, ACSA2)	P56, naïve	Myelin bead removal	3.9 % (ACSA2^+^/CD45^+^/CD11b^+^)
Current study	Unsorted, flow cytometry (CD45, Cd11b, ACSA2)	P56, naïve	Debris removal solution and Myelin bead removal	5.5 % (ACSA2^+^/CD45^+^/CD11b^+^)

**FIGURE 4:**
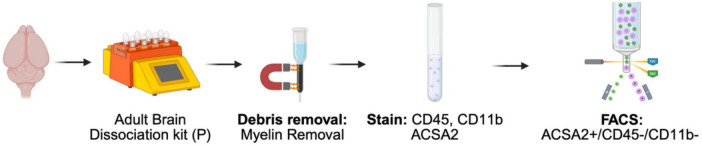
Recommended workflow for ACSA2-based astrocyte isolation with reduced microglial contamination. Adult brain tissue is enzymatically dissociated with the Miltenyi Adult Brain Dissociation kit (P), followed by myelin bead removal to remove myelin debris and reduce microglial contamination. Single-cell suspensions are stained for ACSA2, CD45, and CD11b, then sorted by FACS. Gating on ACSA2^+^/CD45/CD11b cells excludes residual microglia and yields higher purity astrocyte populations. Created in BioRender. Daniele, E. (2026) https://BioRender.com/t52lw3k.

We validated these observations by flow cytometry, confirming that debris removal solution results in significant CD45+/CD11b+contamination (15.2 ± 3.2%), while myelin bead removal reduced this to 3.9% without compromising ACSA2^+^ yield. In contrast, combining debris and myelin removal reduced ACSA2^+^ yield without reducing contamination. Based on these findings, we recommend at minimum using myelin bead removal with additional FACS gating on CD45/CD11b to exclude residual microglia (Figure 4). We further suggest that the purity of cell preparations should be validated by flow cytometry using microglial markers (CD11b, CD45) and astrocyte reporter lines (e.g., Aldh1l1-eGFP).

Microglial contamination in ACSA2-sorted populations lacking adequate debris removal can be further amplified depending on the single cell sequencing platform employed. Scott et al. (2024) observed 53% microglial contamination in the naïve brain by scRNAseq, whereas here, we observed 15% by flow cytometry. Droplet-based platforms, such as 10x Chromium have known biases in cell capture efficiency based on cell size which may lead to differential representation of cell types in the final dataset Yamawaki et al. (2021). Furthermore, flow cytometry quantifies intact cells at the time of sorting, whereas scRNA-seq reflects cells that are retained during encapsulation, potentially introducing discordance between the two levels of analysis. Approaches such as combinatorial barcoding methods [e.g., SPLiT-seq (Rosenberg et al., 2018)] may more accurately reflect the composition of sorted populations.

Overall, our study cautions against use of ACSA2 as a sole astrocyte marker. A limitation of this study is that we cannot comment on age-specific or regional differences in the prevalence of microglia contamination in ACSA2 purified populations given that P56 whole brain tissue was used in our flow cytometry experiments.

## Data Availability

Publicly available datasets were analyzed in this study. This data can be found here: BioProject: PRJNA952594, Runs: SRR27010906 and SRR27010907.

## Footnotes

1
https://gitlab.com/kurogamu/astro_micro_heatmap
