# Supplementary material for: ACSA2 is not astrocyte-specific: implications for cell sorting strategies in the rodent brain
Source: Front Cell Neurosci. 2026 Feb 18;20:1756677. doi: 10.3389/fncel.2026.1756677 (PMC12956691; doi:10.3389/fncel.2026.1756677)
Supplement: Supplementary file 1 [file Data_Sheet_1.docx]

**Supplemental Material**

| **MapMyCells  Annotation** | **Naïve 10X  Chromium cell count** | **Stroke injured 10X  Chromium cell count** |
| --- | --- | --- |
| 317 Astro-CB NN | 0 | 1 |
| 319 Astro-TE NN | 2285 | 755 |
| 324 Microglia NN | 6967 | 5239 |
| 327 Oligo NN | 395 | 581 |
| 330 VLMC NN | 6 | 7 |
| 335 BAM NN | 64 | 69 |
| 333 Endo NN | 59 | 67 |
| 337 DC NN | 28 | 78 |
| 331 Peri NN | 43 | 82 |
| 036 HPF CR Glut | 0 | 1 |
| 314 CB Granule Glut | 0 | 1 |
| 326 OPC NN | 0 | 5 |
| 316 Bergmann NN | 0 | 3 |
| 336 Monocytes NN | 8 | 0 |
| 045 OB-STR-CTX Inh IMN | 1 | 0 |
| **Total** | 9856 | 6889 |

**Supplementary Table 1. MapMyCells cell type classification of ACSA2-sorted samples from naïve and stroke injured brain tissue from Scott *et al.,* 2024**^15^

| **Cell type** | **Gene name** |
| --- | --- |
| Astrocyte markers | Aqp4 |
| Astrocyte markers | Aldoc |
| Astrocyte markers | Gfap |
| Astrocyte markers | Clu |
| Astrocyte markers | Apoe |
| Astrocyte markers | Hes5 |
| Astrocyte markers | Dio2 |
| Astrocyte markers | Agt |
| Astrocyte markers | Sparcl1 |
| Astrocyte markers | Glul |
| Astrocyte markers | Itih3 |
| Astrocyte markers | Atp1b2 |
| Microglia markers | C1qa |
| Microglia markers | C1qb |
| Microglia markers | Ctss |
| Microglia markers | Hexb |
| Microglia markers | P2ry12 |
| Microglia markers | Tyrobp |
| Microglia markers | Trem2 |
| Microglia markers | C1qc |
| Microglia markers | Cd14 |
| Microglia markers | Cx3cr1 |
| Microglia markers | Csf1r |
| Microglia markers | Aif1 |
| Microglia markers | Tmem119 |
| Microglia markers | Itgam |
| Microglia markers | Ptprc |

**Supplementary table 2. Marker genes used for calculation of Seurat Module scores in Figure 1C.**

**
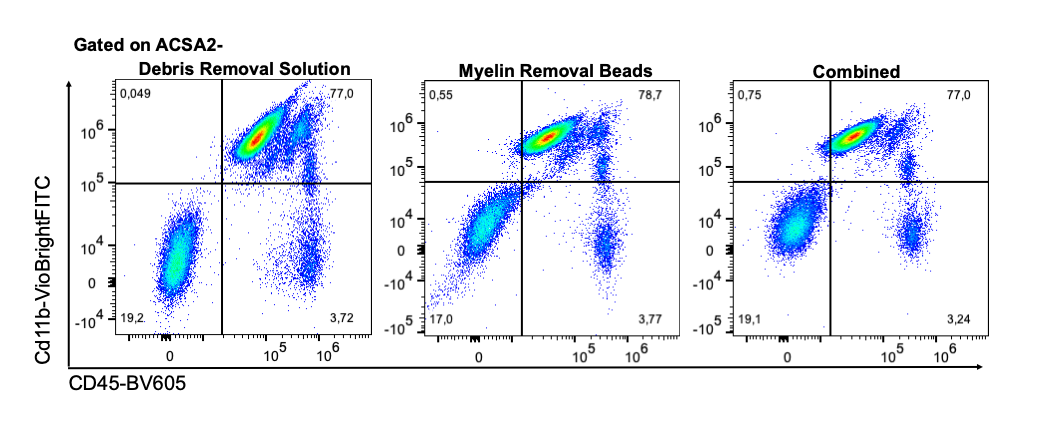
**

**Supplementary Figure 1. CD45+/CD11b+ microglia comprise the majority of ACSA2- fractions regardless of debris removal method.** Representative dot plots showing CD45 and CD11b expression in ACSA2- fractions following debris removal solution, myelin removal beads, or combined debris and myelin removal. (n = 3 per condition).
